# Age and Social Disparities in the Use of Telemedicine During the COVID-19 Pandemic in Japan: Cross-sectional Study

**DOI:** 10.2196/27982

**Published:** 2021-07-23

**Authors:** Atsushi Miyawaki, Takahiro Tabuchi, Michael K Ong, Yusuke Tsugawa

**Affiliations:** 1 Department of Public Health Graduate School of Medicine The University of Tokyo Tokyo Japan; 2 Cancer Control Center Osaka International Cancer Institute Osaka Japan; 3 Division of General Internal Medicine and Health Services Research UCLA David Geffen School of Medicine Los Angeles, CA United States; 4 Department of Health Policy and Management UCLA Fielding School of Public Health Los Angeles, CA United States; 5 VA Center for the Study of Healthcare Innovation, Implementation and Policy Los Angeles, CA United States

**Keywords:** telemedicine, telehealth, disparity, access to care, COVID-19, Japan

## Abstract

**Background:**

The use of telemedicine outpatient visits has increased dramatically during the COVID-19 pandemic in many countries. Although disparities in access to telemedicine by age and socioeconomic status (SES) have been well-documented, evidence is limited as to how these disparities changed during the COVID-19 pandemic. Moreover, the equity of patient access to telemedicine has been scarcely reported in Japan, despite the huge potential for telemedicine expansion.

**Objective:**

We aimed to investigate changes due to age and SES disparities in telemedicine use during the COVID-19 pandemic in Japan.

**Methods:**

Using data from a large internet survey conducted between August 25 and September 30, 2020, in Japan, we examined the associations of participant age and SES (educational attainment, urbanicity of residence, and income level) with their telemedicine use in the following two time periods during the pandemic: April 2020 and August-September 2020.

**Results:**

Of the 24,526 participants aged 18 to 79 years (50.8% [n=12,446] women), the proportion of individuals who reported using telemedicine increased from 2.0% (n=497) in April 2020 to 4.7% (n=1159) in August-September 2020. After adjusting for potential confounders, younger individuals were more likely to use telemedicine than older individuals in April 2020. Although this pattern persisted in August-September 2020, we also observed a substantial increase in telemedicine use among individuals aged 70 to 79 years (adjusted rates, 0.2% in April 2020 vs 3.8% in August-September 2020; *P*<.001 after multiple comparisons). We found disparities in telemedicine use by SES in August-September 2020 that did not exist in April 2020. In August-September 2020, individuals with a university degree were more likely to use telemedicine than those with a high school diploma or less (adjusted rates, 6.6% vs 3.5%; *P*<.001). Individuals living in urban areas exhibited higher rates of telemedicine use than those living in rural areas only in August-September 2020 (adjusted rates, 5.2% vs 3.8%; *P*<.001). Disparities in telemedicine use by income level were not observed in either time period.

**Conclusions:**

In general, younger individuals increased their use of telemedicine compared to older individuals during the pandemic, although individuals in their 70s also increased their use of telemedicine. Disparities in telemedicine use by educational attainment and urbanicity of residence widened during the COVID-19 pandemic.

## Introduction

The COVID-19 pandemic has led to a global public health crisis. More than 81 million cases have been reported worldwide as of the end of December 2020, with 1.8 million deaths from COVID-19 infection [1]. This pandemic has disrupted routine health services across countries, with in-person outpatient visits drastically decreasing in the United States [2,3] and Japan [4]. Telemedicine visits are partially replacing this decline in in-person visits [3,5], and they allow for medical care at a safe social distance in many countries [6]. Changes in the regulations related to the use of telemedicine, including higher reimbursement rates and less restrictive security requirements (eg, allowing non-Health Insurance Portability and Accountability Act–compliant modalities such as Facetime and Skype), facilitated increases in telemedicine usage [6,7].

Telemedicine has the potential to increase access to care for historically underserved populations who experience distance and transportation barriers to the more traditional face-to-face method of providing medical care [8]. Evidence suggests that telemedicine is effective for several types of medical interventions, including smoking cessation [9], psychiatry care [10,11], and management of chronic diseases, such as diabetes, heart disease, and chronic obstructive pulmonary disease [12,13]. Telemedicine can improve health outcomes even among older adults who face additional hurdles when using telemedicine due to sensory barriers and multiple comorbidities [14]. However, there have been concerns that the diffusion of telemedicine technology may have exacerbated social inequality in access to new health care technologies [15]. Racial/ethnic minorities, rural residents, and those with lower educational attainment have relatively limited access to information and communications technologies (ICTs) [16,17], and thus, they are less likely to take advantage of telemedicine. Older adults are also less prepared to use telemedicine than younger adults because of disabilities in hearing and speaking or inexperience with technology [16,18].

Disparities in telemedicine access by age and socioeconomic status (SES) have been well-documented during the COVID-19 pandemic. A study conducted at four clinics in San Francisco reported that the proportion of primary care visits with minority patients aged ≥65 years decreased after telemedicine implementation [19]. Other studies conducted in the United States have found that telemedicine during COVID-19 was concentrated among younger individuals living in urban areas with higher SES [20-23]. Yet, these findings show disparities only at one point in the COVID-19 pandemic, and evidence is limited as to how disparities in telemedicine use by age and SES changed as the need for telemedicine persisted during the COVID-19 pandemic.

Moreover, there is limited research on the sociodemographic patterns in telemedicine use outside the United States. Japan has universal health coverage through the social insurance scheme and is one of the countries with the highest life expectancy in the world [24]. Japan has a lower number of telemedicine users compared to the United States, Canada, and European countries [25]. Recently, mainly due to the impact of the COVID-19 pandemic, policymakers in Japan have been promoting the use of telemedicine. For example, the Japanese government approved the insurance coverage of telemedicine visits in 2018, and further promoted the use of telemedicine during the COVID-19 pandemic. Although the overall use of telemedicine has increased during the COVID-19 pandemic, given that it requires patients to be familiar with health care ICTs, it is possible that it has increased disparity gaps in access to and use of telemedicine between certain subpopulations (eg, old vs young populations). However, the equity of patient access to telemedicine has not been well-characterized in Japan.

To bridge this knowledge gap, we used data from a large internet survey conducted in Japan to examine how disparities in telemedicine use by age and SES may have changed during two time periods following the declaration of the pandemic: April 2020 and August-September 2020. According to the trend in confirmed COVID-19 cases reported in Japan, April 2020 corresponded to the early stage of the first wave of the COVID-19 epidemic (under the state of emergency), and August-September 2020 was during the second wave [1]. The aim of this study was to elucidate how disparities in telemedicine use by age and SES have changed during the pandemic.

## Methods

### Study Design, Setting, and Data Sources

We analyzed data from the *Japan “COVID-19 and Society” Internet Survey (JACSIS)* study [26], a cross-sectional, web-based, self-reported questionnaire survey administered by a major nationwide internet research agency with 2.2 million qualified panelists [27-29]. Selected individuals aged 15 to 79 years (n=224,389) were included in the JACSIS study using stratified random sampling based on gender, age, and prefecture category to represent the distribution of the general population in Japan in 2019 [30,31]. Individuals who agreed to participate in the survey accessed the designated website and responded to questionnaires asking about a wide range of socioeconomic, lifestyle, and health measures in the context of the COVID-19 pandemic. Questionnaires were distributed from August 25, 2020, until September 30, 2020 (hereafter, “August-September 2020”), when the predetermined target number of participants for each gender, age, and prefecture category was met, with an overall response rate of 12.5% (28,000/224,389). For this study, we used a subsample of adults aged 18 to 79 years (n=27,641). We sequentially excluded 2477 individuals showing unnatural or inconsistent responses using the algorithm we developed and 638 individuals whose zip code information was missing. This study was approved by the University of Tokyo (number 2020337NI).

### Exposure Variables

The primary exposure variable was participant age. The secondary exposure variables were SES measures of educational attainment, urbanicity of residence, and household income level. Age was categorized into the following six groups: 18-29 (reference), 30-39, 40-49, 50-59, 60-69, and 70-79 years. Educational attainment was categorized into the following three groups: those who had a university degree or higher (reference; corresponding to the *International Standard Classification of Education* [ISCED] [32] level 6-8), a college degree (ISCED level 5; colleges included junior, community, and vocational colleges), and a high school diploma or lower (ISCED level 0-4). Urbanicity of residence was dichotomized (urban [reference] vs rural). An urban area was defined as a densely inhabited district, which was determined according to the information from the 2015 Census of Japan linked to the seven-digit zip codes reported by the participants [33]; rural area was defined as a nonurban area. Income level was based on self-reported household income and categorized into four groups using the tertiles of household equivalent income (“high” = more than 4.3 million JPY [reference] [110 JPY ≒ 1 USD], “medium” = 2.5 to 4.3 million JPY, or “low” = less than 2.5 million JPY) and an indicator for those who refused to respond to this question.

### Outcome Variables

Our outcome of interest was whether the participant used telemedicine, defined as the examination, diagnosis, and treatment of patients by physicians via information and communication devices that contain real-time visual and auditory information (according to the Ministry of Health, Labour, and Welfare of Japan) [34]. Telemedicine use was measured at the following two different time points during the COVID-19 pandemic in 2020: (1) April and (2) August-September (at the time of the survey). We asked the participants the following question: “Have you ever used telemedicine as a patient?” Participants were to select from one of the following three options: (1) “I have used it before April 2020,” (2) “I used it for the first time after April 2020,” and (3) “Never.” We regarded the participants who chose option 1 as using telemedicine as of April and those who chose option 1 or 2 as using telemedicine as of August-September. We defined the use of telemedicine as those individuals who ever used telemedicine, rather than using the frequency of telemedicine use as the outcome variable, because only survey participants with health issues that require medical attention actually use telemedicine (even those individuals with access to telemedicine would not use telemedicine if they had no health issue during the study period). Moreover, the frequency of telemedicine was affected by not only patients’ access to telemedicine, but also several other factors including the severity of illness of patients and the physicians’ practice patterns (some physicians may see patients more often than others). A similar approach has been used in prior studies [35,36].

### Adjustment Variables

We adjusted for the participants’ sociodemographic and health-related characteristics. The sociodemographic characteristics included employment status (employer, self-employed, employee, and unemployed), marital status (married, never married, widowed, and separated), and household size (number of household members: 1, 2, 3, 4, and 5+). Health-related characteristics included smoking status (never, ever, and current smokers), self-rated health (excellent/good vs moderate/bad/very bad), walking disability (whether the person is experiencing difficulties in walking), and dummy variables for each of nine comorbidities (overweight [BMI ≥25 kg/m^2^] and self-reported presence of eight conditions including hypertension, diabetes, asthma, coronary disease, stroke, chronic obstructive pulmonary disease, cancer, and psychological disorder). BMI was calculated by dividing self-reported body weight (kg) by self-reported body height squared (m^2^).

### Statistical Analysis

First, we described the sociodemographic and health-related characteristics of the participants. To account for the possibility that those who participated and responded to the internet-based survey might differ from the general population, we applied an inverse probability weighting (IPW) approach throughout the analyses [37]. The details of the calculation for IPW are described in Multimedia Appendix 1.

Second, we examined the association between age and rates of telemedicine use in April 2020 or August-September 2020. For each outcome, we constructed a weighted multivariable logistic regression model (IPW described above) that controlled for potential confounders (the other exposures [ie, educational attainment, urbanicity of residence, and income level] and sociodemographic/health-related characteristics). Standard errors were clustered at the prefecture level to account for the potential correlation of participants within the same prefecture. Japan consists of 47 prefectures, which are the country’s first jurisdiction and administrative division levels. To calculate adjusted rates of telemedicine use, we employed marginal standardization (also known as predictive margins or margins of response). For each participant, we calculated predicted probabilities of telemedicine use with the exposure fixed at each category and then averaged over the distribution of the adjustment variables in our sample.

Third, to examine the adjusted change in telemedicine use from April 2020 to August-September 2020, we calculated the difference in the adjusted rates of telemedicine use between these two time points for each age group.

Finally, we tested if the adjusted changes in telemedicine use from April 2020 to August-September 2020 varied across age, by calculating the difference in differences. We also used a similar approach to examine the adjusted rates of telemedicine use and their changes by each SES aspect (educational attainment, urbanicity of residence, and income level).

We used the Benjamini-Hochberg method to account for the multiple comparisons across 15 (6+3+2+4) exposure categories (11 [5+2+1+3] categories for the difference in the adjusted differences), and reported both unadjusted and adjusted *P* values (adjusted *P* values <.05 were considered as statistically significant) [38,39]. All analyses were conducted using Stata version 15 (StataCorp LLC).

### Secondary Analyses

We conducted sensitivity analyses. First, we additionally adjusted for indicator variables for each prefecture (prefecture fixed effects) to effectively compare individuals living in the same prefecture. Second, we additionally adjusted for four categorical variables representing availability of ICTs, including internet access at home and ownership of personal computers, smartphones, and tablet computers, to test whether online access could explain the observed disparities in the rate of telemedicine use by age and SES.

## Results

### Characteristics of the Participants

A total of 24,526 participants (88.7% of the total adult subsample) were included in our analyses. There were 12,446 women (50.8%), and the mean age at the time of the survey was 50.1 (SD 16.6) years. Those who had a high school diploma or less were the most numerous, accounting for half of the participants (12,030/24,526, 49.1%), while one-third (7915/24,526, 32.3%) of the participants had a university degree or higher, and less than 20% (4581/24,526, 18.7%) had a college degree. Approximately 60% (14,666/24,526, 59.8%) of the participants lived in urban areas (Table 1).

**Table 1:** Characteristics of the participants.

Characteristics		Value (N=24,526)
Female, n (%)		12,446 (50.8%)
Age (years), mean (SD)		50.1 (16.6)
**Educational attainment, n (%)**		
	University degree or higher	7915 (32.3%)
	College degree^a^	4581 (18.7%)
	High school diploma or lower	12,030 (49.1%)
**Urbanicity of residence, n (%)**		
	Urban	14,666 (59.8%)
	Rural	9560 (40.2%)
**Income level, n (%)**		
	High	5458 (22.3%)
	Medium	6814 (27.8%)
	Low	7151 (29.2%)
	Not answered	5103 (20.8%)
**Employment status, n (%)**		
	Employer	1011 (4.1%)
	Self-employed	1892 (7.7%)
	Employee	12,623 (51.5%)
	Unemployed	9000 (36.7%)
**Marital status, n (%)**		
	Married	16,102 (65.7%)
	Never married	5413 (22.1%)
	Widowed	1606 (6.6%)
	Separated	1403 (5.7%)
**Household size, n (%)**		
	1	4152 (16.9%)
	2	8386 (34.2%)
	3	5547 (22.6%)
	4	4124 (16.8%)
	≥5	2317 (9.5%)
**Smoking status, n (%)**		
	Never	12,186 (49.7%)
	Ever	7423 (30.3%)
	Current	4917 (20.0%)
Self-rated good health^b^, n (%)		9839 (40.1%)
Walking disability, n (%)		3142 (12.8%)
**Comorbidities, n (%)**		
	Overweight	5202 (21.2%)
	Hypertension	5086 (20.7%)
	Diabetes	1911 (7.8%)
	Asthma	1450 (5.9%)
	Coronary disease	923 (3.8%)
	Stroke	459 (1.9%)
	Chronic obstructive pulmonary disease	332 (1.4%)
	Cancer	774 (3.2%)
	Psychological disorder	1848 (7.5%)

^a^College includes junior, community, and vocational college.

^b^Self-rated good health was defined as very good or good using the 5-point Likert scale question, which asked if self-rated health status was very good, good, moderate, bad, or very bad.

### Overall Trend in the Rates of Telemedicine Use

Of the 24,526 participants aged 18 to 79 years (50.8% [n=12,446] women), the proportion of individuals who reported using telemedicine increased from 2.0% (n=497) in April 2020 to 4.7% (n=1159) in August-September 2020.

### Adjusted Rates of Telemedicine Use by Age

**Figure 1:**
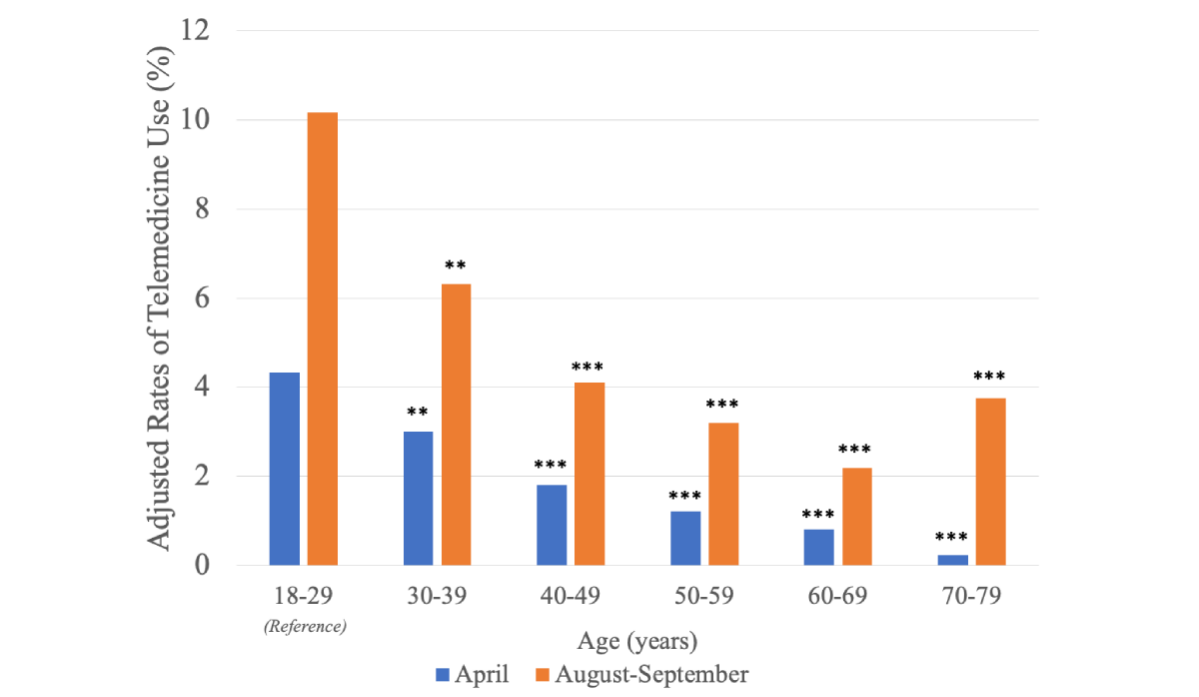
Adjusted rates of telemedicine use in April 2020 and August-September 2020 by age. Telemedicine use was defined as "ever use" of telemedicine at a given time point (April or August-September 2020). Rates of telemedicine use were adjusted for other exposures and sociodemographic/health-related characteristics. We statistically compared the adjusted rates of telemedicine by age groups (reference: age 18-29 years) in April 2020 and in August-September 2020. **Adjusted *P*<.01, ***Adjusted *P*<.001.

After adjusting for potential confounders, younger individuals were more likely to use telemedicine than older individuals in April 2020 (Figure 1 and Multimedia Appendix 2). Although this pattern remained largely unchanged in August-September 2020, with participants aged 18 to 29 years exhibiting the largest increase in telemedicine use (adjusted rates, 4.3% in April vs 10.2% in August-September; adjusted difference, +5.8 percentage points; adjusted *P*<.001), we also observed a substantial increase in telemedicine use among participants aged 70 to 79 years (from 0.2% to 3.8%; +3.5 percentage points; *P*<.001) (Table 2). The increase in the adjusted rates of telemedicine use among participants aged 18 to 29 years was 3.5 percentage points larger compared to that among those aged 40 to 49 years (*P*=.04), 3.9 percentage points larger compared to that among those aged 50 to 59 years (*P*=.01), and 4.5 percentage points larger compared to that among those aged 60 to 69 years (*P*=.003). However, we found no evidence that the increase in the rates of telemedicine use differed between those aged 18 to 29 years and 70 to 79 years (*P*=.19).

**Table 2:** Difference in the adjusted rates of telemedicine use between April 2020 and August-September 2020 by age.

Age group (years)	Number of participants	Adjusted rate, %		Difference^a^, % (95% CI)	*P* value^b^	Difference in differences^a^ (95% CI)	*P* value^b^
		April 2020	August-September 2020				
18-29	3388	4.3	10.2	5.8 (3.0 to 8.7)	<.001	Reference	N/A^c^
30-39	3784	3.0	6.3	3.3 (1.7 to 4.9)	<.001	−2.5 (−5.8 to 0.7)	.19
40-49	4883	1.8	4.1	2.3 (1.2 to 3.3)	<.001	−3.5 (−6.6 to −0.5)	.04
50-59	4278	1.2	3.2	2.0 (0.9 to 3.1)	<.001	−3.9 (−6.9 to −0.8)	.01
60-69	4286	0.8	2.2	1.4 (0.4 to 2.4)	.004	−4.5 (−7.5 to −1.4)	.003
70-79	3907	0.2	3.8	3.5 (2.2 to 4.8)	<.001	−2.3 (−5.5 to 0.8)	.19

^a^We calculated the differences in the adjusted rates of telemedicine use between April 2020 and August-September 2020 for each age group. Then, we examined how the difference in the rates of telemedicine use between the two time points varied by age (difference in differences). The analyses were weighted to account for selection in an internet survey. For each analysis, standard errors were clustered at the prefecture level.

^b^The *P* values were adjusted post hoc to account for multiple comparisons with the use of the Benjamini-Hochberg method.

^c^N/A: not applicable.

### Adjusted Rates of Telemedicine Use by SES Measures

#### Educational Attainment

**Figure 2:**
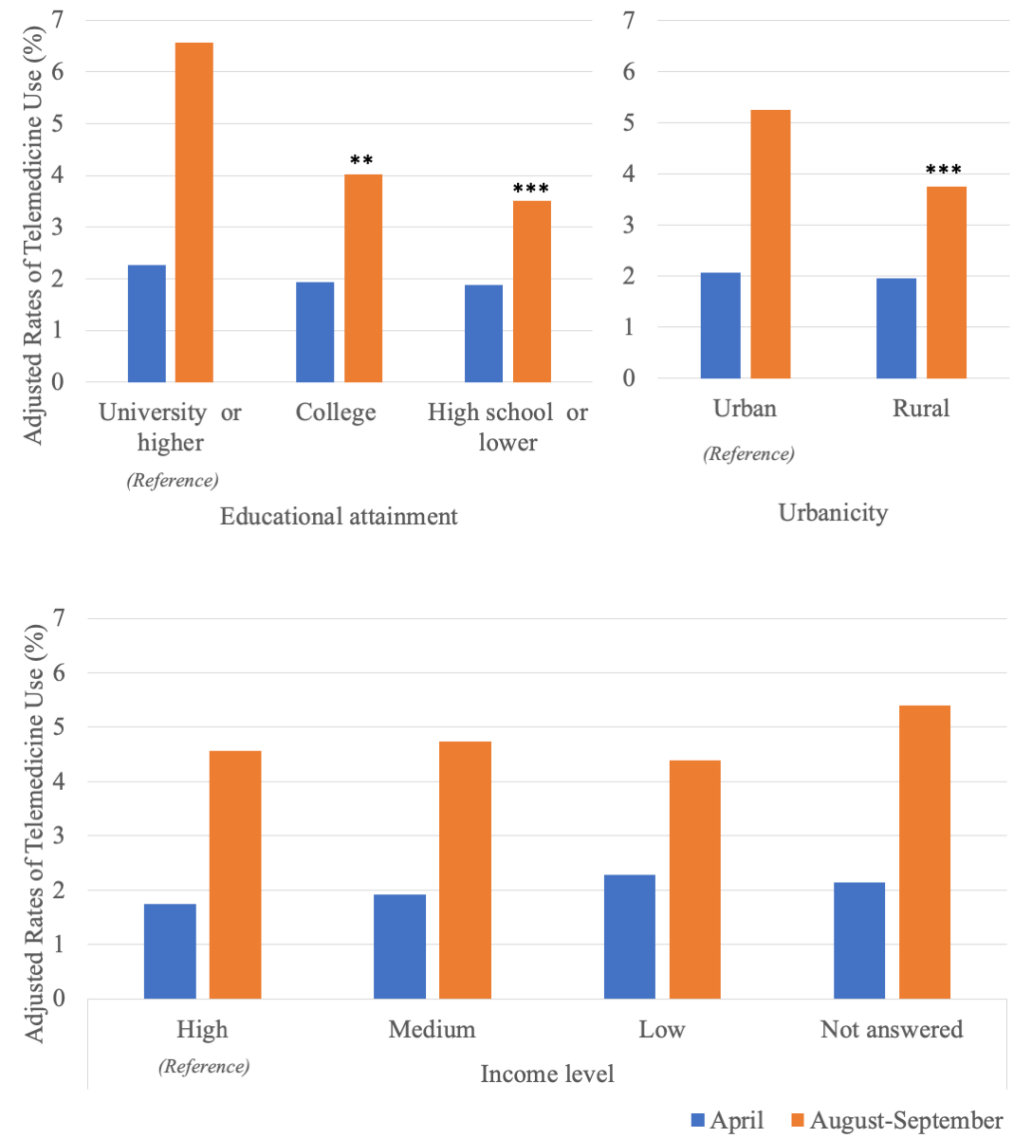
Adjusted rates of telemedicine use in April 2020 and August-September 2020 by socioeconomic status measures. Telemedicine use was defined as "ever use" of telemedicine at a given time point (April or August-September 2020). Rates of telemedicine use were adjusted for other exposures and sociodemographic/health-related characteristics. We statistically compared the adjusted rates of telemedicine by educational attainment (reference: having a university degree or more), urbanicity of residence (reference: living in urban areas), or income level (reference: high income) in April 2020 and in August-September 2020. **Adjusted *P*<.01, ***Adjusted *P*<.001.

We found no evidence that the adjusted rates of telemedicine use differed by educational attainment in April 2020 (Figure 2). In contrast, we found disparities in telemedicine use by SES in August-September 2020. Participants with a university degree or higher were more likely to use telemedicine than those with a college degree (adjusted rates, 6.6% vs 4.0%; *P*=.006) or high school diploma or less (adjusted rates, 6.6% vs 3.5%; *P*<.001) (Multimedia Appendix 2). The increase in the adjusted rates of telemedicine use among participants with a university degree or higher was 2.2 percentage points larger compared to that among those with a college degree (*P*=.01) and 2.7 percentage points larger compared to that among those with a high school diploma or less (*P*=.003) (Table 3).

**Table 3:** Difference in the adjusted rates of telemedicine use between April 2020 and August-September 2020 by socioeconomic status measures.

Measure		Number of participants	Adjusted rate, %		Difference^a^, % (95% CI)	*P* value^b^	Difference in differences^a^ (95% CI)	*P* value^b^
			April 2020	August-September 2020				
**Educational attainment**								
	University or higher	7915	2.3	6.6	4.3 (2.7 to 5.9)	<.001	Reference	N/A^c^
	College	4581	1.9	4.0	2.1 (1.3 to 2.9)	<.001	−2.2 (−4.0 to −0.4)	.01
	High school or lower	12,030	1.9	3.5	1.6 (0.9 to 2.4)	<.001	−2.7 (−4.4 to −0.9)	.003
**Urbanicity of residence**								
	Urban	14,666	2.1	5.2	3.2 (2.4 to 3.9)	<.001	Reference	N/A
	Rural	9860	2.0	3.8	1.8 (1.2 to 2.4)	<.001	−1.4 (−2.3 to −0.4)	.004
**Income level**								
	High	5458	1.7	4.6	2.8 (1.8 to 3.8)	<.001	Reference	N/A
	Medium	6814	1.9	4.7	2.8 (1.5 to 4.2)	<.001	0 (−1.6 to 1.7)	.99
	Low	7151	2.3	4.4	2.1 (1.3 to 2.9)	<.001	−0.7 (−2.0 to 0.6)	.34
	Not answered	5103	2.1	5.4	3.2 (1.1 to 5.4)	.003	0.4 (−2.0 to 2.8)	.79

^a^We calculated the differences in the adjusted rates of telemedicine use between April 2020 and August-September 2020 for each socioeconomic status measure (educational attainment, urbanicity of residence, or income level). Then, we examined how the difference in the rates of telemedicine use between the two time points varied by educational attainment, urbanicity of residence, or income level (difference in differences). The analyses were weighted to account for selection in an internet survey. For each analysis, standard errors were clustered at the prefecture level.

^b^The *P* values were adjusted post hoc to account for multiple comparisons with the use of the Benjamini-Hochberg method.

^c^N/A: not applicable.

#### Urbanicity of Residence

We found no evidence that the adjusted rates of telemedicine use differed by urbanicity of residence in April 2020 (Figure 2). Participants living in urban areas exhibited higher rates of telemedicine use than those living in rural areas only in August-September 2020 (adjusted rates, 5.2% vs 3.8%; *P*<.001) (Multimedia Appendix 2). The increase in the adjusted rates of telemedicine use among participants living in urban areas was 1.4 percentage points higher compared to that among those living in rural areas (*P*=.004) (Table 3).

#### Income Level

We found no evidence that the adjusted rates of telemedicine use differed by income level in April or August-September 2020 (Figure 2). We also found no evidence that the increase in the rates of telemedicine use varied by income level (Table 3).

### Secondary Analyses

Our findings were qualitatively unaffected by additional adjustment for prefecture fixed effects (Multimedia Appendix 3). When we additionally adjusted for the variables representing the availability of ICTs, our findings of increased disparities by SES (ie, university or higher vs high school or lower and urban vs rural) were largely unaffected (Multimedia Appendix 4). However, we no longer found any evidence that the increase in the rates of telemedicine use varied between young individuals (aged 18-29 years) and middle-aged individuals (aged 40-49, 50-59, or 60-69 years), indicating that the trends of the disparities in telemedicine use by age were partly driven by differences in online access.

## Discussion

### Principal Findings

Using data from a large nationwide internet survey conducted in Japan, we found that younger individuals increased the use of telemedicine more than older individuals (leading to wider disparities by age) during the COVID-19 pandemic, although individuals in their 70s also increased the use of telemedicine. Disparities in telemedicine use by educational attainment and urbanicity of residence also increased during the pandemic, whereas we found no evidence that disparities by income level changed.

Younger people were already more likely to use telemedicine as of April 2020; this disparity by age further increased as of August-September 2020 among adults aged 69 years or younger. However, telemedicine use also increased among older adults aged 70 to 79 years, narrowing the gap in telemedicine use among this population. In April 2020, there was no evidence that telemedicine use varied across educational attainment and urbanicity of residence, but by August-September 2020, better educated individuals living in urban areas were more likely to use telemedicine. In contrast, we did not find significant disparities by income level in either April or August-September 2020. Taken together, these findings suggest that improved access to telemedicine during the pandemic may have penetrated unevenly in the population, leaving behind some socioeconomically disadvantaged populations. In contrast, the growing use of telemedicine among older adults was reassuring, indicating that this population of people, who have high health care needs but are often unfamiliar with ICTs [16], benefitted from improved access to telemedicine.

There may be several mechanisms through which the disparities in telemedicine use due to age have widened, especially among individuals aged 69 years or younger. First, younger people may have been more familiar with ICTs, and therefore, faced lower psychological or technological hurdles to initiating telemedicine usage when access to it improved in response to the COVID-19 pandemic in Japan [16]. This explanation is supported by our findings showing no evidence of disparities in telemedicine use due to age once we adjusted for the indicators of ICT availability. Second, the health care needs of young patients may be milder than those of older patients, making young patients more suited to the use of telemedicine. For example, young patients may have fewer comorbidities and seek care for milder conditions compared to middle-aged or older adults. Young patients also may be less likely to need blood tests or diagnostic imaging, which require patients to physically visit a health care facility [40,41]. Finally, younger individuals may be less likely to have disabilities and sensory barriers that could be hurdles to telemedicine use [14].

We also found that individuals aged 70 to 79 years experienced a large increase in telemedicine use. Given that these older adults are in the highest-risk age group for COVID-19 infection (most likely to experience severe life-threatening conditions when infected) [42], they might be incentivized to use telemedicine to avoid visiting health care facilities, which might lead to COVID-19 infection.

The limited increase in telemedicine use for individuals with lower academic attainment may be due to their lower digital literacy, limited access to ICTs, and less flexible work schedules [43], all of which could be barriers to using telemedicine [16]. The limited penetration of telemedicine use in rural areas may be explained by inadequate internet access in rural areas [16] and the fact that there were fewer medical institutions offering telemedicine in rural areas [44]. Given that the number of COVID-19 infections was generally higher in urban areas compared with rural areas, another potential explanation for the relative expansion in telemedicine use in urban areas may be that patients living in urban areas were more incentivized to avoid face-to-face encounters. However, we found that the inclusion of prefecture fixed effects (effectively comparing urban vs rural areas within the same prefecture) did not qualitatively change our results, indicating that this does not fully explain the disparities in telemedicine use between residents of urban and rural areas. It was reassuring that we found no substantial disparities in access to telemedicine by income level throughout the study period. Our findings may indicate that, at least in Japan, financial barriers have minimally influenced the disparities in telemedicine use.

### Comparison With Prior Work

Our findings add to the body of work investigating the impact of age and SES on telemedicine use during the COVID-19 pandemic. Studies that were conducted prior to the expansion of telemedicine use in response to the COVID-19 pandemic have reported that younger patients are more likely to use telemedicine, whereas these studies showed mixed results regarding the disparities by area income level and urbanicity of residence. Studies that were conducted after the COVID-19 pandemic found that telemedicine use overall [41,45,46] and for geriatric care [21], primary care [19,22,47], or otolaryngological care [23] was concentrated among younger individuals living in urban high-income areas. Yet, the timeframe used in these studies represented only one early point in the COVID-19 pandemic, and the studies did not focus on the change over time of age and social disparities in telemedicine use. These previous studies used area income level as an indicator of income level, as opposed to individual income level used in our research. The inconsistent results for income level may partly be attributable to this difference. More importantly, to our knowledge, our study is the first to show that telemedicine use increased substantially among individuals aged 70 years or older, reducing telemedicine disparities among the population at the highest risk for COVID-19 infection.

### Limitations

Our study has some limitations. First, as with any observational study, we could not fully account for unmeasured confounders. Our study also was unable to identify the exact mechanisms of the association between age or SES and the increase in the rates of telemedicine use. Second, due to a limitation in our data, we were unable to identify whether telemedicine use represented telephone visits or virtual visits. Third, there is potential for recall bias; younger individuals with higher SES might be more likely to recall and report telemedicine use. Fourth, we could not identify the clinical conditions for which patients received care via telemedicine. The reports submitted to the government by medical institutions offering telemedicine indicated that the most common conditions for which patients used telemedicine between July and September 2020 were acute relatively mild conditions, such as upper respiratory tract infection, fever, bronchitis, and rhinitis [44]. However, evidence suggests that telemedicine is suitable for psychiatric care [10,11] and management of chronic conditions [12,13]. This gap may be due to patients avoiding clinic visits with signs of infection and shifting to telemedicine, or the Japanese authorities’ restriction on the prescription of psychiatric drugs in telemedicine [44]. The patterns of telemedicine use by age and SES may change as the profiles of conditions treated via telemedicine increase in the future. Finally, because our study sample was collected through an internet-based survey, our findings may not be generalizable to a population with limited access to and/or literacy of the internet. However, we used weighted analyses to minimize the difference in demographics, SES, and health-related characteristics between respondents of the current internet survey and the nationally representative survey, thus approximating our estimates to national estimates.

### Conclusions

Using a large-scale nationwide internet survey in Japan, we found that younger individuals were generally more likely to increase telemedicine use than older individuals during the pandemic, although individuals in their 70s exceptionally gained access to telemedicine. Disparities in telemedicine use by educational attainment and urbanicity of residence increased during the COVID-19 pandemic. These findings indicate that the current telemedicine expansion may be leaving a portion of the socioeconomically disadvantaged population behind, and suggest further need for policy efforts to achieve equal access to health care.

## Appendix

Multimedia Appendix 1 Inverse probability weighting.

Multimedia Appendix 2 Adjusted odds ratios of telemedicine for age and socioeconomic status measures.

Multimedia Appendix 3 Difference in adjusted rates of telemedicine use between April 2020 and August-September 2020 by age and socioeconomic status measures, additionally adjusting for prefecture fixed effects.

Multimedia Appendix 4 Difference in adjusted rates of telemedicine use between April 2020 and August-September 2020 by age and socioeconomic status measures, additionally adjusting for availability of information and communication technologies.

## Abbreviations

ICT: information and communications technology
IPW: inverse probability weighting
ISCED: International Standard Classification of Education
JACSIS: Japan “COVID-19 and Society” Internet Survey
SES: socioeconomic status
