# Supplementary material for: Age and Social Disparities in the Use of Telemedicine During the COVID-19 Pandemic in Japan: Cross-sectional Study
Source: J Med Internet Res. 2021 Jul 23;23(7):e27982. doi: 10.2196/27982 (PMC8315162; doi:10.2196/27982)
Supplement: Multimedia Appendix 1 [file jmir_v23i7e27982_app1.docx]

**Multimedia Appendix 1.** Inverse probability weighting.

The current study applied an inverse probability weighting (IPW) approach throughout the analyses in order to account for the possibility that those who participated and responded to the internet-based survey might differ from the general population.

Internet surveys have several advantages over traditional surveys. However, the potential disadvantage is that they may not be representative of the population of interest because subpopulations with internet access may be specific. Previous studies have used inverse probability weighting (IPW) (derived from propensity scores calculated by a logistic regression model using basic demographic and socio-economic factors such as education and length of home-ownership) obtained from an internet-accessible convenience sample and the nationally-representative sample. It has been suggested that the parameter estimates calculated using IPW are similar, or at least less different, than the population-based estimates [1].

In the current study, we used a population-based sample representative of the Japanese population from the 2016 Comprehensive Survey of Living Conditions (CSLC) to correct for sample selectivity in the internet survey. The CSLC has been conducted every three years by the Japanese Ministry of Health, Labour and Welfare (MHLW) and collects information on health-related factors, such as self-rated health and smoking behavior [2]. Out of inhabited census tracts (sampling unit for the national census in 2010), 5410 census tracts were randomly sampled across Japan in 2016 to collect data from all household members within each census tract. Data were available for 224,208 households (response rate; 77.5%) in 2016. Data from the 2016 CSLC were used because the 2019 CSLC was not yet available at the time of analysis. Data were used with permission from MHLW. CSLC has been used in several studies [3-5].

We pooled and combined data from the two surveys (the current internet survey and CSLC) and ran a multivariable logistic regression model to estimate the probability of "being an internet survey respondent," or propensity score. Propensity scores were calculated for each group stratified by gender and age (15-19, 20-29, ..., 70-79) (gender x age stratification = 14 strata). We used variables available in both surveys (the current internet survey and CSLC) as covariates for the models. For men and women aged 20-79 years, we included socio-economic status (residence area, marital status, educational attainment, and home-ownership) and health-related characteristics (self-rated health and smoking status) in the model. For men and women aged 15-19 years, we included socio-economic status (residence area, educational attainment, and home-ownership) and self-rated health in the model, because they were too young to have a different distribution of marital status, and the CSLC did not ask teenagers about their smoking status. Here, the residential area was categorized into 11 Regions. Marital status was categorized into four groups (married, never married, widowed, and divorced). Education attainment was categorized into five groups (less than high school diploma, high school diploma, college degree, university degree, and master’s or doctor’s degree). Home-ownership was dichotomized. Self-rated health was categorized into five groups based on the 5-point Likert scale (very good,” “good,” “moderate,” “bad,” or “very bad). Smoking status was categorized into three groups (never, ever, and current smokers). A standardized weight was used to keep the total number of respondents included constant.

**References**

1. Schonlau M, van Soest A, Kapteyn A, Couper M. Selection bias in web surveys and the use of propensity scores. Sociological Methods & Research. 2009;37(3):291–318.
2. Ministry of Health, Labour and Welfare. Comprehensive Survey of Living Conditions. 2019 [cited 2021 Jan 1]. URL: https://www.mhlw.go.jp/toukei/list/20-21.html
3. Shibuya K, Hashimoto H, Yano E. Individual income, income distribution, and self rated health in Japan: cross sectional analysis of nationally representative sample. BMJ. 2002 January 5;324(7328):16.
4. Fu R, Noguchi H, Kawamura A, Takahashi H, Tamiya N. Spillover effect of Japanese long-term care insurance as an employment promotion policy for family caregivers. J Health Econ. 2017;56:103–12.
5. Miyawaki A, Kobayashi Y, Noguchi H, Watanabe T, Takahashi H, Tamiya N. Effect of reduced formal care availability on formal/informal care patterns and caregiver health: a quasi-experimental study using the Japanese long-term care insurance reform. BMC Geriatr. 2020 Dec;20(1):207.
