# Supplementary material for: Age and Social Disparities in the Use of Telemedicine During the COVID-19 Pandemic in Japan: Cross-sectional Study
Source: J Med Internet Res. 2021 Jul 23;23(7):e27982. doi: 10.2196/27982 (PMC8315162; doi:10.2196/27982)
Supplement: Multimedia Appendix 2 [file jmir_v23i7e27982_app2.docx]

**Multimedia Appendix 2.** Adjusted odds ratios of telemedicine for age and socioeconomic status measures**.**

|  | (1) April | | | (2) August-September | | |
| --- | --- | --- | --- | --- | --- | --- |
|  | Adjusted odds ratio  (95% CI) | Unadjusted  *P* value | Adjusted  *P* value | Adjusted odds ratio  (95% CI) | Unadjusted  *P* value | Adjusted  *P* value |
|  |  |  |  |  |  |  |
| **Age (yrs)** |  |  |  |  |  |  |
| 18-29 | Reference |  |  | Reference |  |  |
| 30-39 | 0.56 (0.38, 0.83) | .004 | .009 | 0.49 (0.32,0.76) | .002 | .002 |
| 40-49 | 0.23 (0.14, 0.38) | <.001 | <.001 | 0.26 (0.17 ,0.41) | <.001 | <.001 |
| 50-59 | 0.11 (0.05, 0.25) | <.001 | <.001 | 0.18 (0.10 ,0.33) | <.001 | <.001 |
| 60-69 | 0.05 (0.02, 0.12) | <.001 | <.001 | 0.11 (0.06 ,0.20) | <.001 | <.001 |
| 70-79 | 0.01 (0.00, 0.03) | <.001 | <.001 | 0.23 (0.12, 0.43) | <.001 | <.001 |
| **Socio-Economic Status Measures** | |  |  |  |  |  |
| **Educational Attainment** |  |  |  |  |  |  |
| University or higher | Reference |  |  | Reference |  |  |
| College | 0.72 (0.38, 1.37) | .32 | .39 | 0.48 (0.29, 0.79) | .004 | .006 |
| High school or lower | 0.67 (0.44, 1.02) | .06 | .11 | 0.39 (0.25, 0.60) | <.001 | <.001 |
| **Urbanicity of Residence** |  |  |  |  |  |  |
| Urban | Reference |  |  | Reference |  |  |
| Rural | 0.89 (0.55, 1.42) | .61 | .61 | 0.59 (0.44, 0.79) | <.001 | <.001 |
| **Income Level** |  |  |  |  |  |  |
| High | Reference |  |  | Reference |  |  |
| Medium | 1.23 (0.61,2.49) | .57 | .63 | 1.07 (0.70,1.63) | .77 | .85 |
| Low | 1.79 (0.89,3.62) | .10 | .16 | 0.94 (0.61,1.46) | .79 | .79 |
| Not answered | 1.57 (0.66,3.73) | .30 | .41 | 1.31 (0.65,2.65) | .45 | .55 |

CI: confidence interval. Adjusted odds ratios are shown with 95% confidence intervals. We used a weighted multivariable logistic regression model adjusted for the other exposures, and socio-demographic factors (gender, employment status, marital status, and household size), and health-related factors (smoking status, self-rated health, walking disability, indicators of comorbidities). Standard errors were clustered at the prefecture-level. The *P* values were adjusted post hoc to account for multiple comparisons with the use of the Benjamini-Hochberg method.
