# Supplementary material for: Age and Social Disparities in the Use of Telemedicine During the COVID-19 Pandemic in Japan: Cross-sectional Study
Source: J Med Internet Res. 2021 Jul 23;23(7):e27982. doi: 10.2196/27982 (PMC8315162; doi:10.2196/27982)
Supplement: Multimedia Appendix 3 [file jmir_v23i7e27982_app3.docx]

**Multimedia Appendix 3.** Difference in adjusted rates of telemedicine use between April 2020 and August-September 2020 by age and socioeconomic status measures, additionally adjusting for prefecture fixed effects.

|  | Adjusted rate, %  (95% CI) | | Difference,  (2)-(1), %  (95% CI) | *P* value | | Difference-in-differences  (95% CI) | *P* value | |
| --- | --- | --- | --- | --- | --- | --- | --- | --- |
|  | (1) April | (2) August-September |  | Unadjusted | Adjusted |  | Unadjusted | Adjusted |
|  |  |  |  |  |  |  |  |  |
| **Age** | |  |  |  |  |  |  |  |
| 18-29 | 4.3 | 9.7 | 5.4 (2.9, 7.9) | <.001 | <.001 | Reference |  |  |
| 30-39 | 3.1 | 6.4 | 3.2 (1.9, 4.6) | <.001 | <.001 | -2.1 (-5.0, 0.7) | .13 | .18 |
| 40-49 | 2.0 | 4.3 | 2.3 (1.4, 3.3) | <.001 | <.001 | -3.0 (-5.7, -0.4) | .02 | .04 |
| 50-59 | 1.3 | 3.4 | 2.1 (1.1, 3.1) | <.001 | <.001 | -3.3 (-6.0, -0.6) | .01 | .01 |
| 60-69 | 0.9 | 2.3 | 1.5 (0.5, 2.5) | .003 | .003 | -3.9 (-6.6, -1.2) | .004 | .004 |
| 70-79 | 0.3 | 3.6 | 3.3 (2.2, 4.4) | <.001 | <.001 | -2.1 (-4.8, 0.6) | .13 | .20 |
| **Socio-Economic Status Measures** | | |  |  |  |  |  |  |
| **Educational Attainment** | |  |  |  |  |  |  |  |
| University or higher | 2.4 | 6.4 | 4.0 (3.1, 4.9) | <.001 | <.001 | Reference |  |  |
| College | 2.0 | 4.0 | 2.1 (1.1, 3.1) | <.001 | <.001 | -1.9 (-3.2, -0.6) | .004 | .004 |
| High school or lower | 1.9 | 3.6 | 1.7 (1.2, 2.3) | <.001 | <.001 | -2.3 (-3.3, -1.3) | <.001 | <.001 |
| **Urbanicity of Residence** | |  |  |  |  |  |  |  |
| Urban | 2.2 | 5.2 | 3.0 (2.7, 3.4) | <.001 | <.001 | Reference |  |  |
| Rural | 2.0 | 3.8 | 1.8 (1.5, 2.2) | <.001 | <.001 | -1.2 (-1.7, -0.7) | <.001 | <.001 |
| **Income Level** |  |  |  |  |  |  |  |  |
| High | 1.9 | 4.8 | 2.9 (2.1, 3.7) | <.001 | <.001 | Reference |  |  |
| Medium | 2.0 | 4.7 | 2.7 (1.8, 3.5) | <.001 | <.001 | -0.2 (-1.4, 0.9) | .69 | .76 |
| Low | 2.3 | 4.4 | 2.1 (1.2, 3.0) | <.001 | <.001 | -0.8 (-2.0, 4.0) | .19 | .23 |
| Not answered | 2.2 | 5.3 | 3.1 (1.6, 4.6) | <.001 | <.001 | 0.2 (-1.5, 1.9) | .82 | .82 |

We adjusted for the covariates in the main analyses plus indicator variables for each prefecture (prefecture fixed effects). See Table 2 and Table 3’s legends for more details.
