# Supplementary material for: Age and Social Disparities in the Use of Telemedicine During the COVID-19 Pandemic in Japan: Cross-sectional Study
Source: J Med Internet Res. 2021 Jul 23;23(7):e27982. doi: 10.2196/27982 (PMC8315162; doi:10.2196/27982)
Supplement: Multimedia Appendix 4 [file jmir_v23i7e27982_app4.docx]

**Multimedia Appendix 4.** Difference in adjusted rates of telemedicine use between April 2020 and August-September 2020 by age and socioeconomic status measures, additionally adjusting for availability of information and communication technologies.

|  | Adjusted rate, %  (95% CI) | | Difference,  (2)-(1), %  (95% CI) | *P* value | | Difference-in-differences  (95% CI) | *P* value | |
| --- | --- | --- | --- | --- | --- | --- | --- | --- |
|  | (1) April | (2) August-September |  | Unadjusted | Adjusted |  | Unadjusted | Adjusted |
|  |  |  |  |  |  |  |  |  |
| **Age** | |  |  |  |  |  |  |  |
| 18-29 | 4.1 | 8.7 | 4.6 (1.8, 7.4) | <.001 | <.001 | Reference |  |  |
| 30-39 | 3.0 | 5.6 | 2.6 (1.1, 4.1) | <.001 | <.001 | -2.0 (-5.2, 1.1) | .20 | .31 |
| 40-49 | 1.9 | 3.9 | 2.0 (1.0, 3.0) | <.001 | <.001 | -2.6 (-5.6, 0.3) | .08 | .16 |
| 50-59 | 1.3 | 3.3 | 2.0 (0.9, 3.1) | <.001 | <.001 | -2.6 (-5.6, 0.4) | .08 | .15 |
| 60-69 | 0.9 | 2.5 | 1.6 (0.6, 2.6) | .002 | .002 | -3.0 (-6.0, 0) | .04 | .12 |
| 70-79 | 0.2 | 4.3 | 4.1 (2.7, 5.5) | <.001 | <.001 | -0.5 (-3.6, 2.6) | .74 | .90 |
| **Socio-Economic Status Measures** | | |  |  |  |  |  |  |
| **Educational Attainment** | |  |  |  |  |  |  |  |
| University or higher | 2.3 | 6.4 | 4.2 (2.8, 5.6) | <.001 | <.001 | Reference |  |  |
| College | 1.9 | 4.1 | 2.2 (1.4, 3.1) | <.001 | <.001 | -1.9 (-3.6, -0.3) | .02 | .06 |
| High school or lower | 1.9 | 3.6 | 1.7 (0.9, 2.5) | <.001 | <.001 | -2.5 (-4.1, -0.9) | .002 | .002 |
| **Urbanicity of Residence** | |  |  |  |  |  |  |  |
| Urban | 2.1 | 5.3 | 3.2 (2.5, 3.9) | <.001 | <.001 | Reference |  |  |
| Rural | 2.0 | 3.8 | 1.8 (1.2, 2.4) | <.001 | <.001 | -1.4 (-2.3, -0.4) | .004 | .004 |
| **Income Level** |  |  |  |  |  |  |  |  |
| High | 1.8 | 4.5 | 2.8 (1.8, 3.7) | <.001 | <.001 | Reference |  |  |
| Medium | 1.9 | 4.8 | 3.0 (1.6, 4.3) | <.001 | <.001 | 0.2 (-1.5, 1.9) | .81 | .89 |
| Low | 2.3 | 4.4 | 2.1 (1.3, 3.0) | <.001 | <.001 | -0.6 (-1.9, 0.6) | .31 | .43 |
| Not answered | 2.2 | 5.2 | 3.0 (1.1, 4.9) | .002 | .002 | 0.2 (-1.9, 2.4) | .82 | .82 |

We adjusted for the covariates in the main analyses plus four categorical variables representing availability of information and communications technologies (ICTs), including internet access at home and ownership of personal computers, smartphones, and tablet computers. See Table 2 and Table 3’s legends for more details.
